# Genome-wide investigation of *superoxide dismutase* (*SOD*) gene family and their regulatory miRNAs reveal the involvement in abiotic stress and hormone response in tea plant (*Camellia sinensis*)

**DOI:** 10.1371/journal.pone.0223609

**Published:** 2019-10-10

**Authors:** Chengzhe Zhou, Chen Zhu, Haifeng Fu, Xiaozhen Li, Lan Chen, Yuling Lin, Zhongxiong Lai, Yuqiong Guo

**Affiliations:** 1 College of Horticulture, Fujian Agriculture and Forestry University, Fuzhou, Fujian, China; 2 Institute of Horticultural Biotechnology, Fujian Agriculture and Forestry University, Fuzhou, Fujian, China; 3 Key Laboratory of Tea Science of Fujian Province, Fujian Agriculture and Forestry University, Fuzhou, Fujian, China; ICAR - National Research Center on Plant Biotechnology, INDIA

## Abstract

Superoxide dismutases (SODs), as a family of metalloenzymes related to the removal of reactive oxygen species (ROS), have not previously been investigated at genome-wide level in tea plant. In this study, 10 *CsSOD* genes were identified in tea plant genome, including 7 *Cu/Zn-SODs* (*CSDs*), 2 *Fe-SODs* (*FSDs*) and one *Mn-SOD* (*MSD*), and phylogenetically classified in three subgroups, respectively. Physico-chemical characteristic, conserved motifs and potential protein interaction analyses about CsSOD proteins were carried out. Exon-intron structures and codon usage bias about *CsSOD* genes were also examined. Exon-intron structures analysis revealed that different *CsSOD* genes contained various number of introns. On the basis of the prediction of regulatory miRNAs of *CsSOD*s, a modification 5’ RNA ligase-mediated (RLM)-RACE was performed and validated that csn-miR398a-3p-1 directly cleaves *CsCSD4*. By prediction of *cis*-acting elements, the expression patterns of 10 *CsSOD* genes and their regulatory miRNAs were detected under cold, drought, exogenous methyl jasmonate (MeJA) and gibberellin (GA_3_) treatments. The results showed that most of *CsSODs* except for *CsFSD2* were induced under cold stress and *CsCSD*s may play primary roles under drought stress; exogenous GA_3_ and MeJA could also stimulated/inhibited distinct *CsSOD*s at different stages. In addition, we found that csn-miR398a-3p-1 negatively regulated the expression of *CsCSD4* may be a crucial regulatory mechanism under cold stress. This study provides a certain basis for the studies about stress resistance in tea plants, even provide insight into comprehending the classification, evolution, diverse functions and influencing factors of expression patterns for *CsSOD* genes.

## Introduction

Tea plant (*Camellia sinensis*) is an important commercial crop and has been cultivated for more than 2000 years in China [1]. In recent years, nevertheless, whose quality and spatial distribution have been constantly affected by several abiotic stresses with the tea-producing regions expanding to the north and the global extreme climate occurring frequently, especially cold and drought [2–3]. According to previous research, cold stress is a serious environmental stress that damage cells and limits plant growth [4], while drought stress reduced tea production and increased tea plant mortality [5]. The most common effects of such stress is the generation of toxic reactive oxygen species (ROS), including superoxide radicals (O^**·**^_2_^-^), hydroxyl radical (OH·) and hydrogen peroxide (H_2_O_2_) [6]. Excessive ROS could result in cell membrane damage, protein oxidation and DNA damage, and eventually lead to metabolic disorder and apoptosis in plants, which has irreversible adverse effects on plant yield. To alleviate the oxidative damage caused by excessive ROS, an efficient and complex antioxidant ROS scavenging system was formed in the long-term process of plant development, including many non-enzymatic and enzymatic defense systems. Superoxide dismutases (SODs), act as the first line of defense in antioxidant system, play a significant role in catalyzing the dismutation of superoxide free radicals and protecting plant cells from oxidative damage [7]. SOD is a family of metalloenzymes related to the removal of ROS in aerobic organisms. Based on the type of metal cofactor, the SODs have been classified into three categories, Cu/Zn-SOD (CSD), Fe-SOD (FSD) and Mn-SOD (MSD), respectively [8]. Moreover, another type of SOD, Ni-SOD (NSD), was also discovered in streptomyces, but have not ever been found in plant [9]. SOD plays an important role in scavenging ROS and maintaining the balance of active oxygen via catalyzes the conversion or dismutation of toxic superoxide anion radicals to synthesize O_2_ and H_2_O_2_ in plants [10]. Under normal circumstance, the synthesis and homeostasis of ROS maintaining a dynamic balance and will not cause damage to plants. However, the dynamic balance will be destroyed under a series of stress. Once the free radical accumulation exceeds the threshold, it will poison the cells [11]. Hitherto, *SOD* genes family have been widely studied in many plant species, including arabidopsis (*Arabidopsis thaliana*) [12], tobacoo (*Nicotiana tabacum*) [13], populus (*Populus trichocarpa*) [14], longon (*Dimocarpus longan*) [15], soybean (*Glycine max*) [16], sorghum (*Sorghum bicolor*) [17], banana (*Musa acuminate*) [18], diploid cotton (*Gossypium raimondii* and *G*. *arboretum*) [19], upland cotton (*G*. *hirsutum*) [20], cucumber (*Cucumis sativus*) [21], foxtail millet (*Setaria italica*) [22], grapevine (*Vitis vinifera*) [9], japanese larch (*Larix kaempferi*) [23], and rapeseed-mustard crops (*Brassica juncea* and *Brassica rapa*) [24], etc. In previous studies, in *A*. *thaliana*, the transcription and translation level of *SOD* genes increased significantly after subjected to oxidative stress [12]; in *G*. *hirsutum*, under drought stress, the expression of three types of *SOD* genes was significantly up-regulated; but under cold stress, only the expression of *CSD* genes were increased, and the photosynthetic rate and soluble sugar were also increased [25]; after transfer *MSD* gene from pea into rice, the drought tolerance was significant enhanced and also exhibited less injury [26]; in tea plant, under the cold stress, the content of ROS was higher together with lower SOD activity, and the leaf damage was more serious in the cold-sensitive cultivar compared to the cold-resistant cultivar [27]. Thus, increasing the activity of SOD is one of the effective ways for plants to resist a series of abiotic stress.

MicroRNAs (miRNAs), a group of 20–24 nt in length, single-stranded non-coding RNAs that negatively regulate gene expression at the post-transcriptional levels [28]. Evidence is accumulating that miRNAs play key roles in the regulation of abiotic responses in plant. For example, miR398 negatively regulate the expression level of *CSDs* by cold and oxidative stress in *A*. *thaliana* [29]; miR165/166 involved in drought resistance each in *A*. *thaliana* [30] and *O*. *sativa* [31]. In 2010, 13 miRNAs were computational identified. Among them, 11 miRNAs targeted to 37 potential targets in tea plant [32–33]. This was the first finding for the prediction and analysis of tea plant miRNAs through bioinformatics tools, and it provide insight into understanding the function and processing of tea miRNAs. Subsequently, there were also many miRNAs have been found that related to stress resistance in tea plant, such as miR156, miR166a, and miR398 [2–3]. However, the regulatory miRNAs of *CsSOD* genes are still unknown.

To date, there was no research about the identification and analysis of *SOD* genes at genome-wide level in tea plant. It is possible that identify all of the *SOD* family members at genome-wide level with the genome data of tea plant (cultivar ‘Yunkang 10’ and cultivar ‘Shuchazao’) were made available to the public [34–35]. In this study, we performed a research to identify and analyze all the members of *SOD* in tea plant, including physico-chemical characteristics, phylogenetic relationships, exon-intron structure, motif composition, potential protein interaction, and codon usage bias (CUB). By using miRNA database from previous published article [3], we predicted the regulatory miRNAs of *CsSOD* genes, then, the modification 5’ RNA ligase-mediated (RLM)-RACE was carried out to verify the putative cleavage sites of *CsSODs*. Moreover, the *cis*-acting elements of *CsSODs* promoters involved in stress responses and hormone stimuli were also predicted to further clarify the regulatory mechanisms of Cs*SOD*s expression. Refer to the predicted results, we investigated the expression patterns of the *CsSOD* genes and their regulatory miRNAs under cold, drought, exogenous gibberellin (GA_3_), and methyl jasmonate (MeJA) treatments via quantitative real-time polymerase chain reaction (qRT-PCR). This systematic study about *CsSOD* gene family will provide a certain basis for the studies about exploration of *CsSODs* diverse functions.

## Materials and methods

### Retrieval of *SOD* genes in tea plant

Known AtSOD amino acid sequences downloaded from NCBI as baits, the local BLASTP was executed to search the SOD proteins in two tea plant genomes (cultivar ‘Yunkang 10’ and cultivar ‘Shuchazao’). Functional annotations were filtered for Pfam identifiers of the SOD domains (PF00080, PF00081, and PF02777). Then, the filtered sequences were uploaded to Simple Modular Architecture Research Tool (SMART) (http://smart.embl-heidelberg.de/) [36] and Conserved Domain Database (CCD) (https://www.ncbi.nlm.nih.gov/cdd/) [37], respectively, to further verify the accuracy of these sequences. Information about the length of CDS and amino acid sequences of CsSOD was obtained from the tea plant genome database [34–35]. The WoLF PSORT web server (https://wolfpsort.hgc.jp/) [38] and ProtParam tool in ExPASy web (http://www.expasy.org/) [39] were used to predict the subcellular localizations and the physico-chemical characteristics of CsSOD proteins, respectively.

### Phylogenetic tree construction

The full-length of SOD protein sequences from monocotyledonous plant (*O*. *sativa* and *M*. *acuminata*), and dicotyledonous plant (*A*. *thaliana*, *C*. *sinensis*, *D*. *longan*, and *P*. *trichocarpa*) were aligned using the Muscle program with default parameters [40]. For step further classification, we constructed a phylogenetic tree using MEGA5.1 software with the neighbor-joining (NJ) algorithm and setting 1000 bootstrap replicates, other parameters were all default [41]. Then, using iTOL web tool (https://itol.embl.de/) [42] to visualize the phylogenetic tree.

### Analysis of exon-intron structure and conserved motifs

A separate clustering diagram for CsSOD and AtSOD proteins was built by MEGA 5.1 software [41]. The exon-intron structures of *SOD* genes from tea plant and *A*. *thaliana* were identified via upload the Genetic Feature Format (GFF3) file from tea plant and *A*. *thaliana* genome data to TBtools program for analysis [43]. The sequences of CsSOD and AtSOD proteins were analyzed by MEME web server (http://meme-suite.org/tools/meme) [44] to predicted conserved motifs. Selecting “5 motifs should MEME find” mode and other parameters were left as default. Then, using TBtools program to combine the results of phylogenetic tree, exon-intron structures and conserved motifs from tea plant and *A*. *thaliana* into one map.

### Analysis of potential protein interaction

The SOD protein interaction network was constructed by the STRING 11.0 (https://string-db.org/cgi/input.pl) [45] on the basis of the orthologs in known AtSOD. Setting the network edges as confidence, the parameters as medium confidence parameter (0.400) and no more than 10 interactors to show.

### Analysis of codon usage bias about *CsSOD* genes

The measured parameters of CUB, including ENC, RSCU, CAI, GC and GC3s were calculated by CodonW program [46], and the data processed in Excel 2013.

### Prediction of regulatory elements of *CsSOD* genes

For *cis*-acting elements prediction, we extracted the upstream genome sequence within 2000 bp of the start codon from each *CsSOD* gene as putative promoter region, then using the PlantCARE serve (http://bioinformatics.psb.ugent.be/webtools/plantcare/html/) [47] to predicted *cis*-acting elements. *CsSOD* genes targeted by miRNAs were predicted via searching the CDS sequences of all *CsSOD* genes for complementary sequences of the tea plant miRNAs using the psRNATarget (https://plantgrn.noble.org/psRNATarget/analysis?function=3) [48] with default parameters, and any targets with an overall score below 5.0 was considered to be a credible miRNA target. The miRNA database was from previous published article [3].

### Tea plant materials and experimental treatments

This experiment employed 2-year-old seedlings of tea plant cultivar ‘Tieguanyin’, which were cultivated in a conservatory belong to college of horticulture of Fujian Agriculture and Forestry University, Fuzhou, China (26°05′N, 119°18′E). The conservatory is a place for teaching and scientific research, so, there were no specific permissions were required, and this experiment did not involve any endangered or protected plant materials. The conservatory ambient conditions maintain 25°C ± 2°C temperature, 14/10 h light/dark photoperiod, and 70% relative humidity. Then, those seedlings were subject to different treatments, including cold and drought, and hormone treatments (MeJA and GA) refer to previous reported procedures with minor revise [49–50]. Before these processing, the second leaves from the seedlings were collected at “0 h” as CK. For cold treatments, the temperature in the conservatory was modulated to 4°C and other conditions remain unchanged. For drought treatments, the seedling was irrigated with 15% (w/v) PEG 4000 to simulate drought stress. For exogenous MeJA and GA_3_ treatments, freshly prepared working solutions of 1 mmol/L MeJA and GA_3_ were sprayed on 2 different seedlings, respectively. Then, second tender leaves from each seedling were sampled after 12 hours, 24 hours, 36 hours, and 48 hours, respectively. Then, frozen all the samples in liquid nitrogen and stored at −80°C immediately. Three independent biological replicates were employed.

### RNA isolation, primers designing, and qRT-PCR analysis

Total RNA from all the samples were isolated using Transzol up (TransGen, Beijing, China). The integrity of the isolated RNA samples was detected by 0.8% agarose gel electrophoresis, and purity and concentration were tested by NanoDrop 2000 Spectrophotometer (Thermo Fisher Scientific, Waltham, USA). Those samples whose OD_260_/OD_280_ ratios between 1.90 and 2.10 were selected for reverse transcribe to cDNA for *CsSOD* genes and first-strand cDNA of miRNA using the Prime-Script^™^ Reagent Kit (Takara, Otsu, Japan) and TransScript miRNA First-strand cDNA Synthesis Super Mix (TransGen, Beijing, China), respectively.

The *GAPDH* (accession no. GE651107) and *β-Actin* (accession no. LOC114296039) genes in tea plant were both used as reference genes [51] for *CsSOD*s, while U6 and 5.8s were both used as internal references for regulatory miRNAs of *CsSODs*. Specific primers of *CsSOD* genes and reference genes (S5 Table) for qRT-PCR system were designed and verified using tea plant information archive (TPIA) platform [52] and DNAMAN 9 software, respectively, while the miRNA universal primer was from TransScript miRNA First-strand cDNA Synthesis Super Mix (TransGen, Beijing, China), and the specific primers of miRNA and their internal references were shown in S5 Table. The qRT-PCR detection about *CsSOD* genes and their regulatory miRNAs under different treatments were conducted on LightCycler 480 (Roche Applied Sciences, Basel, Switzerland) platform using Hieff qPCR SYBR Green Master Mix (Yeasen, Shanghai, China) and TransStart Tip Green qPCR SuperMix (TransGen, Beijing, China), respectively. Relative expression level was calculated by 2^−ΔCt^ method [53]. Statistical analyses were conducted using SPSS 25 software, and the data were analyzed by one-way analysis of variance followed by Tukey’s post-hoc test [51].

### Cleavage site identification with modified 5’ RLM-RACE

To verified the miRNA cleavage sites of *CsCSD4*, *CsCSD7* and *CsFSD2* respectively, a modified 5’ RLM-RACE experiment was performed using First-Choice RLM-RACE Kit (Thermo Fisher Scientific, carlsbad, USA) with the manufacturer’ s instructions. In brief, total RNA (5 μg) from equal mixtures of all the RNA samples were ligated to the adapter for 5’ RACE without treatment with dephosphorylation and decapped. The first round PCR amplification of cDNA fragments was performed using the 5’ RACE outer primer from the manufacturer and gene-specific outer primer (after putative complementary regions of miRNAs about 200 bp) (S5 Table). Then, the first round PCR products were used as the template of nested PCR with 5' RACE inner primer from the manufacturer and gene-specific inner reverse primers (S5 Table). Finally, the resulting PCR fragments were gel-purified by EasyPure Quick Gel Extraction Kit (TransGen, Beijing, China), then cloned into pMD 18-T Vector (Takara, Otsu, Japan) for sequencing (BioSune, Fuzhou, China).

## Results

### A total of 10 *SOD* genes were identified in tea plant

In this study, we searching the tea plant genome database (cultivar ‘Yunkang 10’ and cultivar ‘Shuchazao’) using Pfam identifiers of CSD domain (PF00080) and FSD / MSD domain. Both FSD and MSD had the FSD/MSD N-terminal domain (PF00081) and the FSD/MSD C-terminal domain (PF02777). Finally, we both ascertained a total of 10 *SOD* genes in two tea plant genomes, of which, 7 members belong to CSDs, 2 members belong to FSDs, and only one member was MSD. Based on their scaffold ID order and Pfam types, we named these 10 *CsSOD* genes as *CsCSD1* to *CsCSD7*, *CsFSD1* to *CsFSD2* and *CsMSD1*, respectively. The gene names, transcript IDs, scaffold IDs, coding sequences (CDS), and amino acid sequences were listed in S1 Table. After performed BLASTP on NCBI, we found that the matching rate of part CsSODs in cultivar ‘Shuchazao’ genome (TEA012288, TEA005110, TEA023332, TEA012017) were low compared to known *CsSOD* genes (only 68%, 25%, 33%, and 54%, respectively). Thus, the subsequent analyses all use CsSOD sequences in cultivar ‘Yunkang 10’ genome.

The physico-chemical characteristics of identified CsSOD proteins analyses, including the lengths, molecular weights (MWs), isoelectric points (pIs), instability index, grand average of hydropathicity (GRAVY), aliphatic index, and subcellular prediction of CsSOD proteins were showed in Table 1. All the 10 CsSOD proteins ranged from 139 (CsCSD5) to 333 (CsFSD1) aa in length, and have MWs from 14.20 (CsCSD5) to 37.42 (CsFSD1) kDa, pIs from 4.90 (CsCSD6) to 7.14 (CsFSD2), instability index from 14.34 (CsCSD5) to 42.49 (CsFSD2), GRAVY from -0.546 (CsFSD1) to 0.326 (CsCSD6), and aliphatic index from 69.79 (CsFSD1) to 92.36 (CsCSD7), respectively. The pI values showed that all members of CsSOD except CsFSD2 were acidic. The values of instability index < 40 or > 40 are determine the protein will be stable in a test tube or not [54]. Other than CsFSD2, most CsSOD proteins were predicted to be stable. According to the result of putative subcellular localization, CsCSD1, CsCSD2, CsCSD3, CsCSD5, CsCSD6 and CsFSD2 proteins were localized in cytoplasm while CsCSD4, CsCSD7 and CsFSD1 proteins were in chloroplasts; and CsMSD1 protein was in mitochondrion (Table 1). The information of previous studies about SOD subcellular localization [55] corroborated our findings.

**Table 1:** Physico-chemical characteristics of identified CsSOD proteins.

Name	Protein(aa)	MW(kDa)	pI	Instability index	GRAVY	Aliphatic index	Subcellular location
CsCSD1	143	24.03	6.39	22.49	-0.196	84.56	Cytoplasm
CsCSD2	204	21.16	5.72	20.64	0.116	90.29	Cytoplasm
CsCSD3	152	15.47	5.62	18.22	-0.135	81.97	Cytoplasm
CsCSD4	219	22.62	6.82	21.17	-0.045	88.72	Chloroplast
CsCSD5	139	14.20	5.91	14.34	-0.188	76.40	Cytoplasm
CsCSD6	249	25.71	4.90	20.19	0.326	86.10	Cytoplasm
CsCSD7	208	21.49	6.44	17.71	-0.080	92.36	Chloroplast
CsFSD1	333	37.42	5.42	35.36	-0.546	69.79	Chloroplast
CsFSD2	243	28.26	7.14	42.49	-0.516	79.05	Cytoplasm
CsMSD1	205	22.41	6.22	30.25	-0.201	90.93	Mitochondrion

### Phylogenetic classification of SOD proteins in different plants

**Fig 1:**
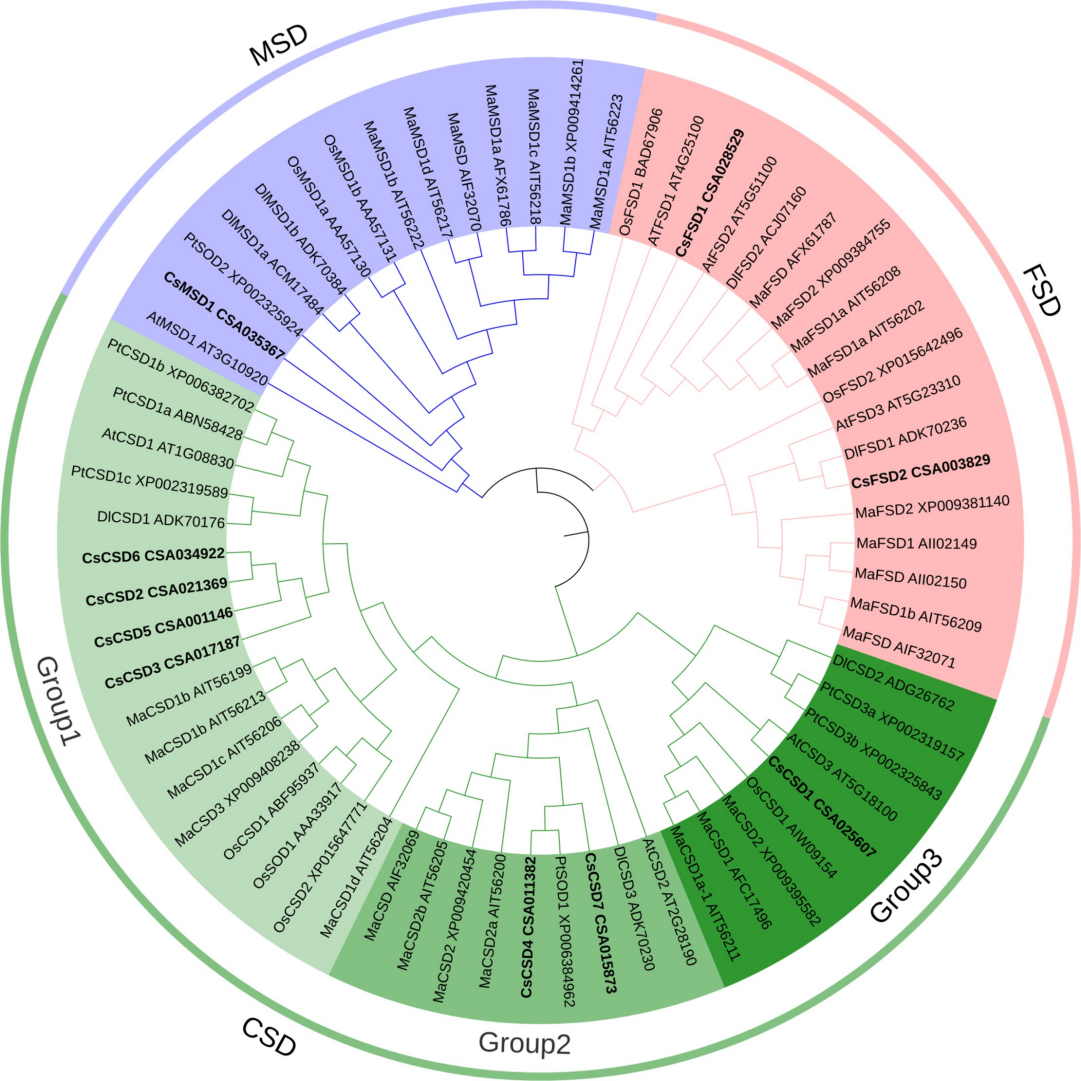
Phylogenetic tree of SOD proteins from *O*. *sativa*, *M*. *acuminata*, *A*. *thaliana*, *D*. *longan*, *P*. *trichocarpa*, and *C*. *sinensis*. Os: *Oryza sativa*; Ma: *Musa acuminata*; At: *Arabidopsis thaliana*; *Dl*: *Dimocarpus longan*; Pt: *Populus trichocarpa*; Cs: *Camellia sinensis*.

To investigate the evolutionary relationship of SOD in different plant species, we aligned and constructed a phylogenetic tree based on full-length of SOD protein sequences from monocotyledonous plants, *O*. *sativa* and *M*. *acuminata*, and dicotyledonous plants, *C*. *sinensis*, *A*. *thaliana*, *D*. *longan* and *P*. *trichocarpa* (Fig 1). The result showed that all the SOD proteins from different plant species were clustered into three major categories, which in line with their metal cofactor types, Cu/Zn- (CSD), Fe- (FSD) and Mn- (MSD), respectively. Further analysis, MSDs and FSDs were highly similar, and belong to the same subgroup, while the CSDs were quite different from them. The CSD proteins of each species gathered together and showed cluster distribution, indicating that CSDs were highly conserved in different plants. Notably, CsCSD1 and AtCSD3, CsCSD4 and PtSOD1 were in a smallest clade respectively, further indicate that CSD proteins were highly conserved in dicotyledon.

To step further analysis of clustering result, CSD proteins were subdivided into 3 subgroups (Group1, Group2 and Group3). CsCSD2, CsCSD3, CsCSD5 and CsCSD6 proteins were in Group 1; CsCSD4 and CsCSD7 proteins were in Group 2; and CsCSD1 protein was in Group 3. It was also found in Group 1 that CSD members from *O*. *sativa* and *M*. *acuminata* were clustered into a small branch, while the CSD members from tea plant, *A*. *thaliana*, *D*. *longan* and *P*. *trichocarpa* were in another small branch. It is speculated that there may be some different evolution strategies in CSD protein sequences between monocotyledonous and dicotyledonous plants.

### Exon-intron structures and conserved Motifs of CsSOD and AtSOD

**Fig 2:**
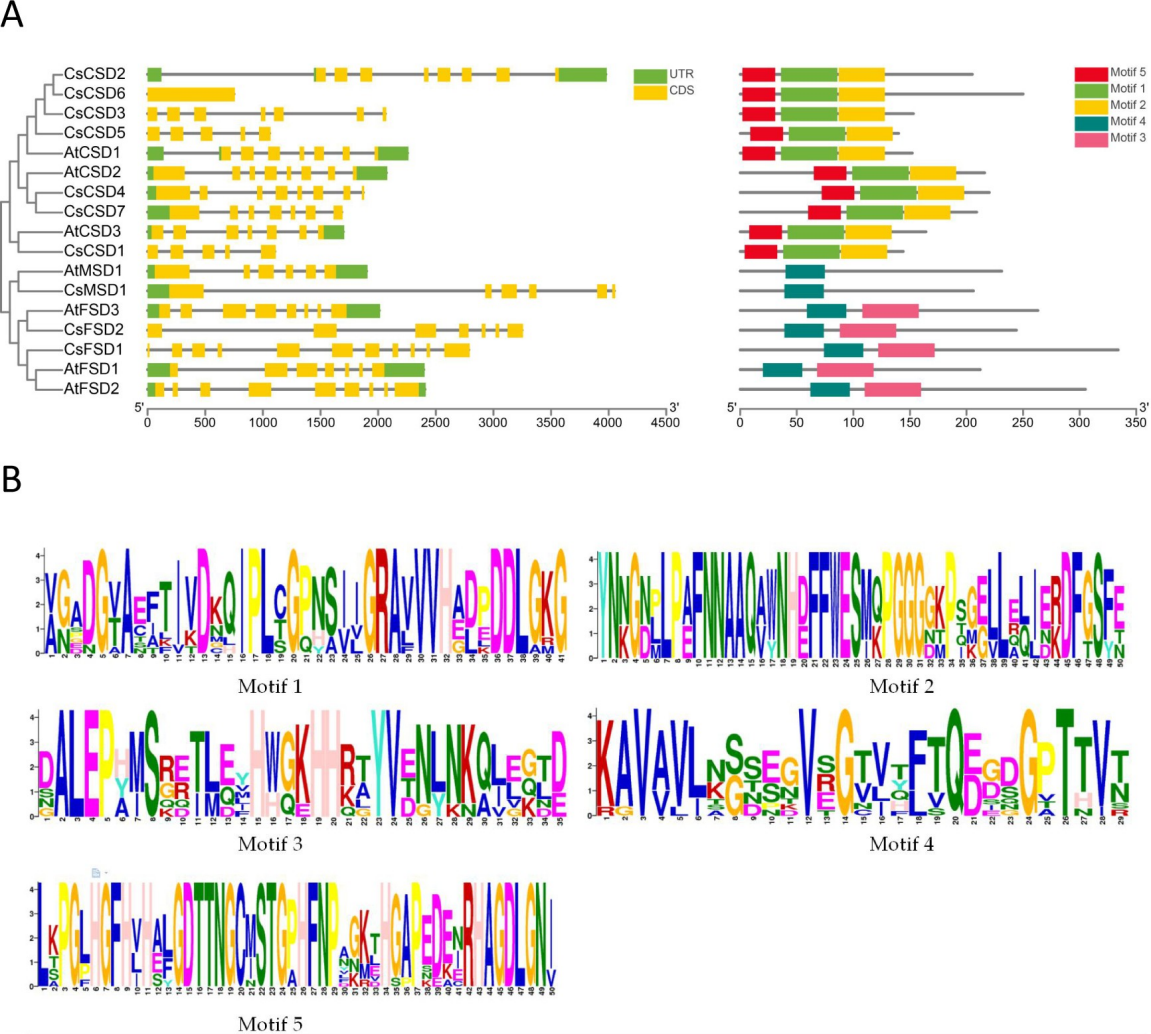
Phylogenetic tree, conserved motifs, and motif logos of CsSOD and AtSOD proteins and exon-intron structures of *CsSOD* and *AtSOD* genes. (A) Phylogenetic tree, exon-intron structures, conserved motifs of CsSOD and AtSOD proteins; (B) Motif logos of CsSOD and AtSOD proteins.

A separate cluster of SOD proteins from *C*. *sinensis* and *A*. *thaliana* (CsSOD and AtSOD) was shown in Fig 2A. To step further clarify the structure features of *C*s*SOD*s, a comparative analysis of exon-intron structure was executed between *CsSOD* and *AtSOD* genes (Fig 2A). The *CsSOD* genes exhibited different exon-intron organizational patterns and not in accord with phylogenetic clustering. *CsSOD* genes contained introns from 0 (*CsCSD2*) to 10 (*CsFSD1*) and *AtSOD* genes contained introns from 5 (*AtMSD1*) to 8 (*AtFSD2*). Compare to *AtSOD*, the number of intron varies greatly among different *CsSOD* genes. Further analysis, all exon-intron junction sites of *CsSOD* genes were spliced consistent with the eukaryotic GT-AG splice rules [56].

Furthermore, we also searched for the presence of conserved motifs in all members of CsSOD proteins compare to *A*. *thaliana* (Fig 2B). Motif 1, 2 and 5 were found in all CSD proteins while all FSD proteins possess motif 3 and 4. Both CsMSD1 and AtMSD1 proteins contain only motif 4 (Fig 2A). According to the results of CDD annotation, Motif 1, 2 and 5 were all belong to “Cu_Zn Superoxide Dismutase superfamily”. Motif 3 and 4 were both belong to “Sod_Fe_C superfamily”. Different motifs were specifically present in CSD, FSD and MSD, respectively, in accord with their phylogenetic clustering.

### Potential CsSOD protein–protein interaction

**Fig 3:**
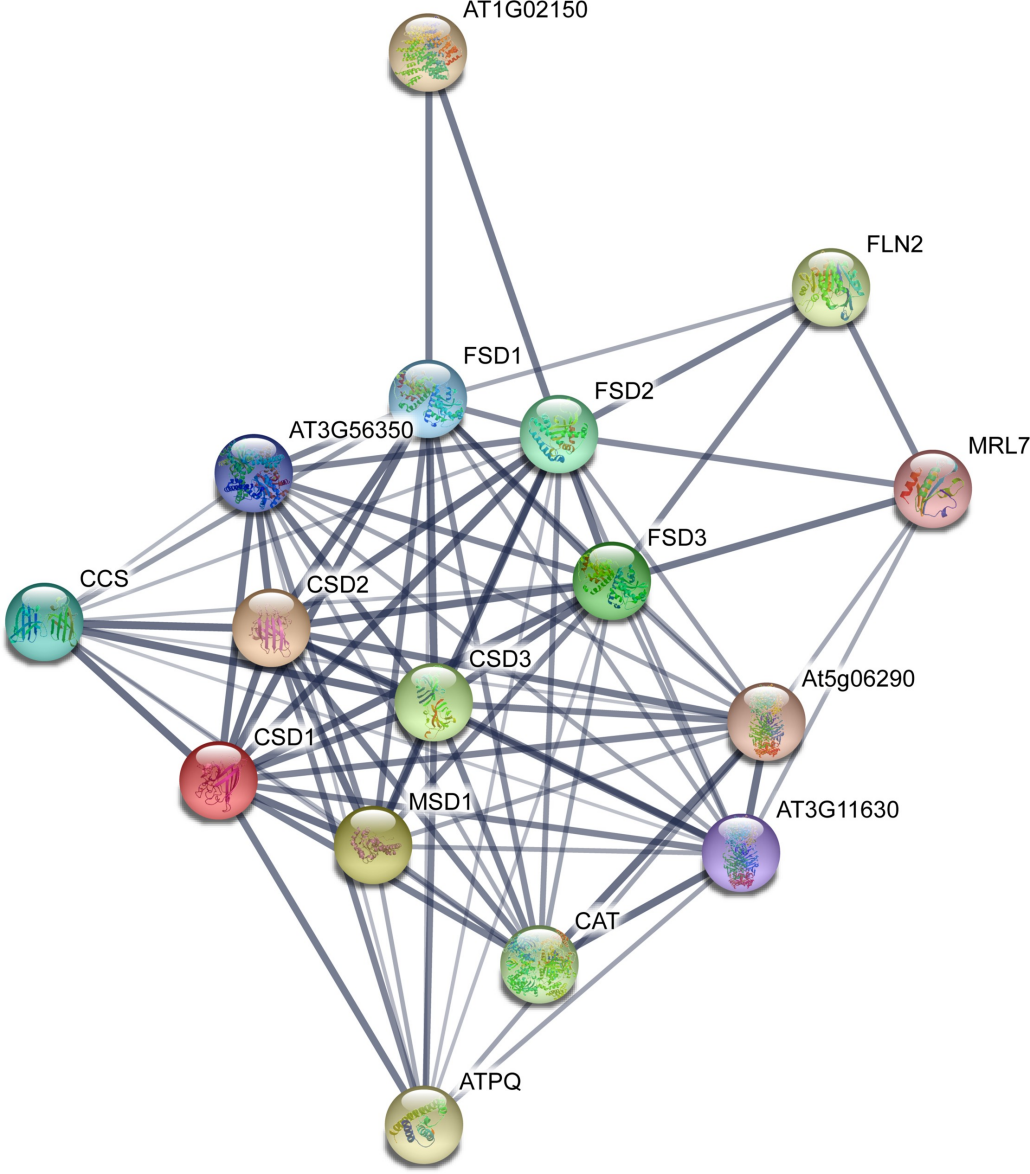
Potential protein–protein interaction network of CsSODs. CsCSD2, CsCSD3, CsCSD5, and CsCSD6 were displayed as homologous CSD1 protein in *A*. *thaliana*; CsCSD4 and CsCSD7 were displayed as homologous CSD2 in *A*. *thaliana*; and CsCSD1 was displayed as homologous CSD3 in *A*. *thaliana*; CsFSD1 and CsFSD2 were displayed as homologous FSD2 and FSD3 proteins in *A*. *thaliana*, respectively. And CsMSD1 was displayed as homologous MSD1 protein in *A*. *thaliana*. The higher the interaction coefficient, the thicker the line between proteins, vice versa.

On the basis of the orthologs in *A*. *thaliana*, the potential CsSOD protein–protein interaction was analyzed. The homologous AtSOD proteins with the highest identity were regarded as “STRING proteins”. As shown in Fig 3, all the 10 CsSOD proteins associated with 6 known AtSOD proteins in the interaction network. For CSDs, CsCSD2, CsCSD3, CsCSD5, and CsCSD6 in connection with AtCSD1; CsCSD4 and CsCSD7 associated with AtCSD2; and AtCSD3 was the highest homologous protein of CsCSD1. For FSDs, CsFSD1 and CsFSD2 corresponding to AtFSD2 and AtFSD3, respectively. And CsMSD1 associated with AtMSD1. As expected, the corresponding result was exactly same as the phylogenetic classification, which CsSOD proteins corresponding to three types of AtSOD proteins were also divided into same subgroup in the phylogenetic tree (Fig 1). Three types of SOD proteins involved in a strong interaction networks, they may play a regulatory role through the formation of protein complexes. Moreover, CSDs were involved in a strong interaction with copper chaperone for superoxide dismutase (CCS). CCS is responsible for the transfer of Cu^2+^ into cytoplasm, which increases the concentration of Cu^2+^ in the cytoplasm and thereby promoting the expression of CSD1 [57]. In addition, the high level of interaction between CSDs and catalase (CAT) implied various enzymes in plant antioxidant enzyme system might respond to various biological and abiotic stresses by synergetic response.

### Codon usage bias of *CsSOD* genes

The phenomenon of CUB can reflect the origin, evolution and mutation patterns of genes and given an important reference value for analysis of gene function and expression [58–59]. Thus, analysis of CUB patterns could provide necessary theoretical guidance for the further studies of species evolution law, gene function and gene expression. In this study, the values of ENC, CAI, GC and GC3s of *CsSOD* genes were shown in Table 2. We can observe that the ENC values of *CsSOD* genes were between 42.12 and 58.09 and the average value was 50.12, indicating that the codons of *CsSOD* genes have no obvious bias and relatively low expression level in tea plant, same as CAI value validation. The CAI values of *CsSOD* genes varied from 0.19 (*CsFSD1*) to 0.26 (*CsMSD1*), which were far less than 0.5. GC and GC3s analyses showed that the GC values of *CsCSD4* and *CsCSD7* were greater than 0.5, accounting for only 20% of all the subjects studied, and only the GC3s value of *CsMSD1*> 0.5. We calculated that the average value of GC3s was 0.41 at whole-genome scale in tea plant cultivar ‘Yunkang 10’ and cultivar ‘Shuchazao’, meaning that *CsSOD* genes except *CsMSD1* were consistent with tea plant genome on CUB pattern. Moreover, RSCU values of 59 codons in all *CsSOD* genes showed that there were 34 optimal codons, including CUU, GUU, ACC, and AGA in *CsCSD1*; CCU, GCU, and AGG in *CsCSD2*; CUU, GUU, AGC, GCU, and AGG in *CsCSD3*; CUC, UCC, and CCA in *CsCSD4*; CUU, AUU, GUU, ACA, and GCU in *CsCSD5*; CCU in *CsCSD6*; CUC, UCC, and CGU in *CsCSD7*; AGU, CGU, ACA, and AGA in *CsFSD1*; CUU, ACA, and AGA in *CsFSD2*; AGC and CGG in *CsMSD1*, meaning *CsSOD* genes has stronger bias on these codons, and these codons may be the optimal codons of the *CsSOD* genes (S2 Table).

**Table 2:** The values of CAI, ENC, GC, and GC3s of *CsSOD* genes, ‘Yunkang 10’ and ‘Shuchazao’ genome.

Name	CAI	ENC	GC	GC3s
CsCSD1	0.23	50.98	0.49	0.44
CsCSD2	0.24	45.71	0.48	0.39
CsCSD3	0.25	46.48	0.49	0.33
CsCSD4	0.22	49.39	0.52	0.44
CsCSD5	0.23	42.12	0.46	0.27
CsCSD6	0.22	55.95	0.48	0.35
CsCSD7	0.20	49.29	0.51	0.43
CsFSD1	0.19	51.05	0.44	0.32
CsFSD2	0.20	52.16	0.42	0.37
CsMSD1	0.26	58.09	0.50	0.51
‘Yunkang 10’ genome	0.20	51.91	0.45	0.41
‘Shuchazao’ genome	0.20	52.02	0.41	0.44

### Various functions of *cis*-acting elements in *CsSOD* promoters

**Fig 4:**
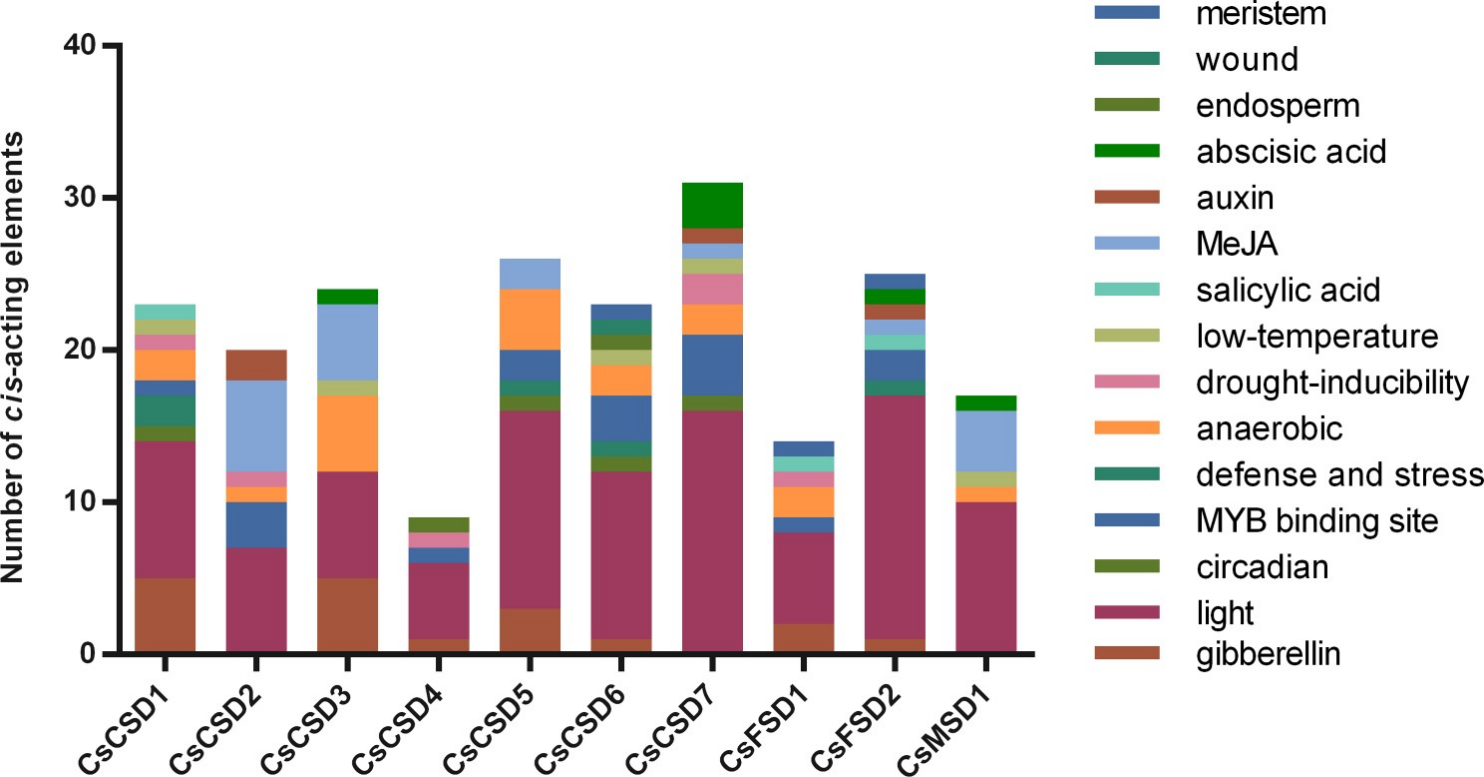
Different *cis*-acting elements in putative CsSOD promoters which associated with abiotic stresses, hormone responses, growth and development.

For seeking *cis*-acting elements of *CsSOD* genes, we extracted the upstream 2000 base pairs (bp) genome sequences of the start codon from each *CsSOD* gene as putative promoter region. We filtered the unknown elements because of the information about promoter sequences of *CsCSD2*, *CsCSD4*, *CsFSD2* and *CsMSD1* were insufficient (existing 710 bp, 1117 bp, 130 bp and 533 bp N-containing sequences, respectively). Thus, we found a total of 34 types *cis*-acting elements about abiotic stress and hormone responses in putative promoters of all *CsSOD* genes (S3 Table). On analysis, CAAT box and TATA box which related to common promoter and enhancer regions and core promoter element of transcription start, respectively, were found in all promoter regions. Moreover, a large number of light-responsive elements were also been found in all promoters and the number varied from 5 (*CsCSD4*) to 16 (*CsCSD7* and *CsFSD2*). Other functions of predicted *cis*-acting elements were showed in Fig 4.

These hormone-responsive elements, including P-box, TATC-box and GARE-motif; TCA-element; CGTCA-motif and TGACG-motif; TGA-element; and ABRE, which associated with GA_3_, salicylic acid (SA), MeJA, Auxin and abscisic acid (ABA) response, respectively. Of which, more than half of members contain GA- and MeJA- responsive elements. For stress-responsive elements, TC-rich repeats; ARE and GC-motif; MYB binding site (MBS); low temperature responsiveness (LTR); and WUN-motif, related to defense and stress, anaerobic induction, drought inducibility, low-temperature inducibility, and wound stress, respectively. Therein, drought inducibility and low-temperature inducibility elements were found in over half of all *CsSOD* members. Some regulatory elements for plant growth and development, including GCN4-motif and CAT-box were allied to endosperm expression and meristem expression have also been found in *CsCSD4*, *CsCSD6*, *CsFSD1 and CsFSD2*.

Such number of *cis*-acting elements in typical *CsSOD* promoter regions manifesting that *CsSOD* genes should be involved in distinct regulatory mechanisms in stress resistance and the growth development in tea plant.

### Validation of csn-miR398a-3p-1 cleavage of *CsCSD4*

**Fig 5:**
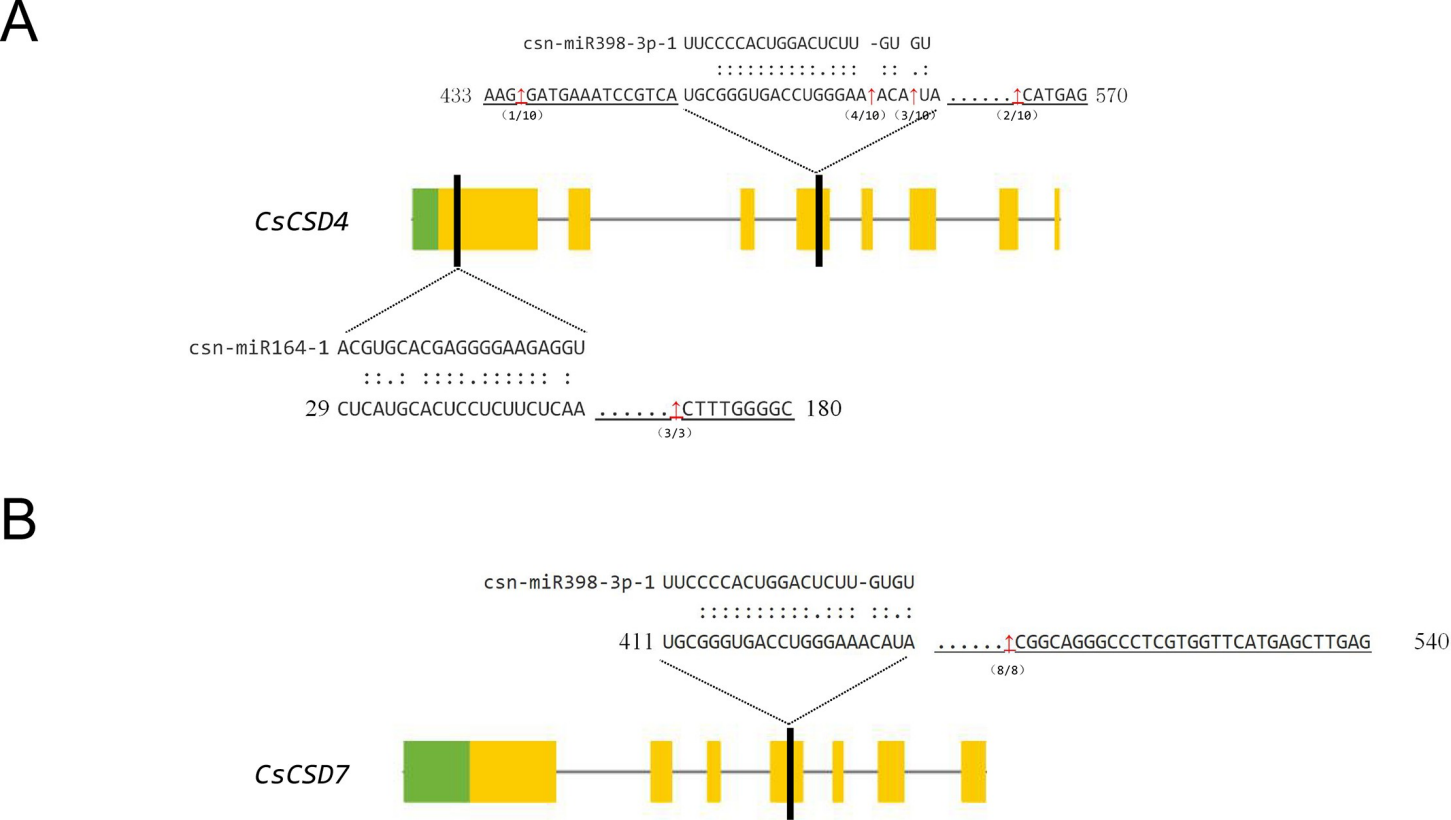
Cleavage sites mapping of *CsCSD4* and *CsCSD7* genes. (A) The cleavage sites mapping of *CsCSD4* was aligned with miR164-1 and csn-miR398a-3p-1. (B) The cleavage site mapping of *CsCSD7* was aligned with csn-miR398a-3p-1. Numbers in bracket indicate the number of cleavage site, and the cleavage sites were displayed by red arrow. Spots between miRNA and target genes represent complementary sequence.

As a class of single-stranded, non-coding small RNAs, miRNAs can suppress the expression of specific target genes by guiding the cleavage of target mRNA or inhibiting them translate into homologous proteins [60]. Hence, predicting the regulatory miRNA of *CsSOD* genes is crucial way for understanding the regulatory factor about *CsSOD* expression pattern at posttranscriptional level. By prediction, five miRNAs (miR398a-3p-1, miR164-1, novel-miR54, miR166-5d-1, and miR159a-1) regulate three *CsSODs* (*CsCSD4*, *CsCSD7* and *CsFSD2*) by triggering the cleavage (S4 Table). To be specific, *CsCSD4* was targeted by csn-miR164-1, and csn-miR398a-3p-1; *CsCSD7* was targeted by csn-miR159a-1, csn-miR398a-3p-1, and novel-miR54; and *CsFSD2* was targeted by csn-miR166d-5p-1. To further identify cleavage sites, fragments of the *CsCSD4*, *CsCSD7*, and *CsFSD2* mRNAs were detected using the modification 5’ RLM-RACE. The electrophoretogram (S2 Fig) showed that the nested PCR productions of *CsCSD4* was between 100 bp and 250 bp, others were all shorter than 100 bp. Sequencing result showed that no cleavage products cloned from the novel-miR54 and miR159a-1 complementary region of *CsCSD7*, and miR166d-5p-1 complementary region of *CsFSD2* were detected. Moreover, the cleavage sites all mapped outside the complementary regions of csn-miR398a-3p-1 and miR164-1 complementary regions in *CsCSD7* and *CsCSD4*, respectively (Fig 5). Most fraction of cleavage products (7/10) cloned from *CsCSD4* were detected between csn-miR398a-3p-1 complementary region (Fig 5A). This result firmly proved that *CsCSD4* is directly cleaved by csn-miR398a-3p-1. The expression of each miRNAs and their target genes need to detected in further experiments to determine how they exert specific functions in tea plant.

### Expression patterns of *CsSOD* genes and their regulatory miRNAs under different treatments

**Fig 6:**
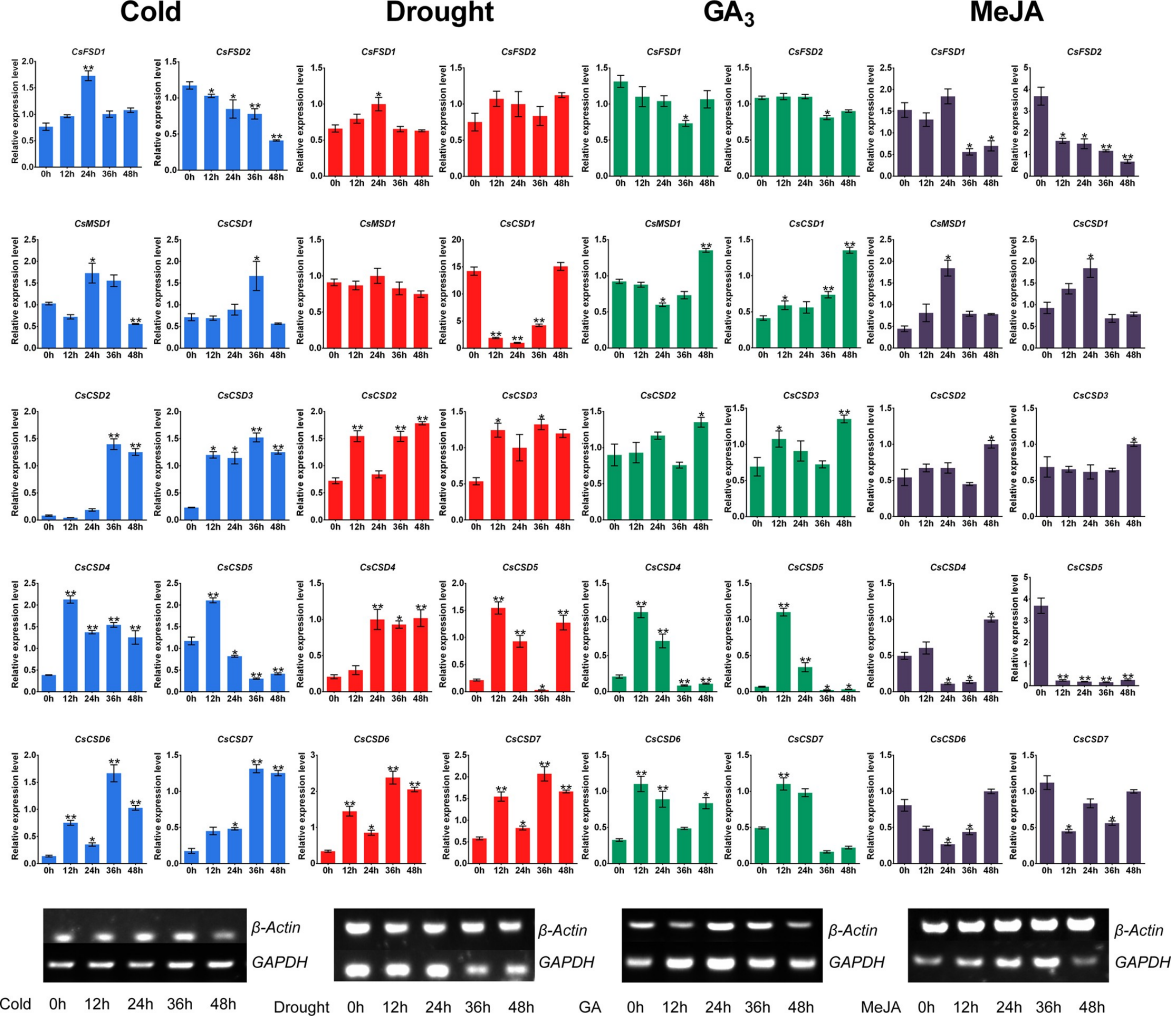
Expression patterns of *CsSOD* genes under cold, drought, exogenous GA_3_ and MeJA treatments in tea plant cultivar ‘Tieguanyin’. The electrophoretograms of *GAPDH* and *β-Actin* under different treatments were showed on the bottom of figure. Data shown were the mean of three independent repeated experiments ± standard deviation (SD), and were analyzed by one-way analysis of variance (ANOVA) followed by Tukey’s post-hoc test. * indicates significant difference (*p*<0.05) and ** indicates extremely significant difference (*p*<0.01).

To clarify the potential roles of *CsSOD* genes involved in abiotic stresses and hormone stimuli, we used qRT-PCR to determine the expression levels of *CsSOD* genes under cold, drought, exogenous GA_3_ and MeJA treatments from 0 h to 48 h (Fig 6 and S6 Table). The expression level at 0 h as the control check (CK).

Treated by cold stress (4°C), all members of *CsSOD* genes were significantly induced or suppressed. Apart from *CsFSD2* was inhibited and its expression bottoming at 48 h, the other 9 *CsSOD* genes expression was up-regulated at different stages, such as the expression of *CsCSD4* and *CsCSD5* peaked at 12 h; *CsFSD1* and *CsMSD1* reached the peak at 24 h; other members but *CsFSD2* were at the highest point at 36 h.

To simulated drought stress, the ‘Tieguanyin’ seedling was irrigated with 15% (w/v) polyethylene glycol 4000 (PEG 4000). Then, we found that the expression trends of *CsFSD1* and *CsCSD1* were just reverse, which revealed “up-down-up” and “down-up-down” trends from 0 h to 48 h, respectively. Furthermore, *CsCSD2*, *CsCSD3*, *CsCSD6*, and *CsCSD7* showed a similar trend of expression, which the expression levels at 24 h were lower than 12 h, 36 h, and 48 h but still higher than 0 h. While the expression of *CsFSD2* and *CsMSD1* were relatively stable.

Treated by exogenous GA_3_ (1 mmol/L), both the expressions of *CsFSD1* and *CsFSD2* were down-regulated at 36 h; *CsMSD1*, *CsCSD1*, *CsCSD2*, and *CsCSD3* were up-regulated and peaked at 48 h; and from 0 h to 12 h, 12 h to 36 h, 36 h to 48 h, both *CsCSD6* and *CsCSD7* showed a “up-down-up” trend. Moreover, *CsCSD4* and *CsCSD5* were highly induced genes, the highest point (12 h) and lowest point (36 h) were over 12-fold and 55-fold, respectively.

Treated by exogenous MeJA (1 mmol/L), we found that both the expressions of *CsMSD1* and *CsCSD1* were significantly increased from 0 h to 24 h then decreased after 24 h, but the expression of *CsCSD6* just opposite; *CsFSD1* and *CsCSD7* also have a similar expression profile but *CsFSD1* still maintained relatively low expression at 48 h; *CsFSD2* and *CsCSD5* were markedly suppressed and maintained relatively low expression levels; *CsCSD2* and *CsCSD3* showed relatively stable; and *CsCSD4* was highly induced gene, its expression was down-regulated from 24 h to 36 h but significantly up-regulated at 48 h.

Taken together, most *CsSOD* genes were significantly induced/repressed by diversiform treatments. It speculated that distinct *CsSOD* members had specific functions and adopt different coping strategies to response to stress or phytohormone signal at different stages in tea plant.

**Fig 7:**
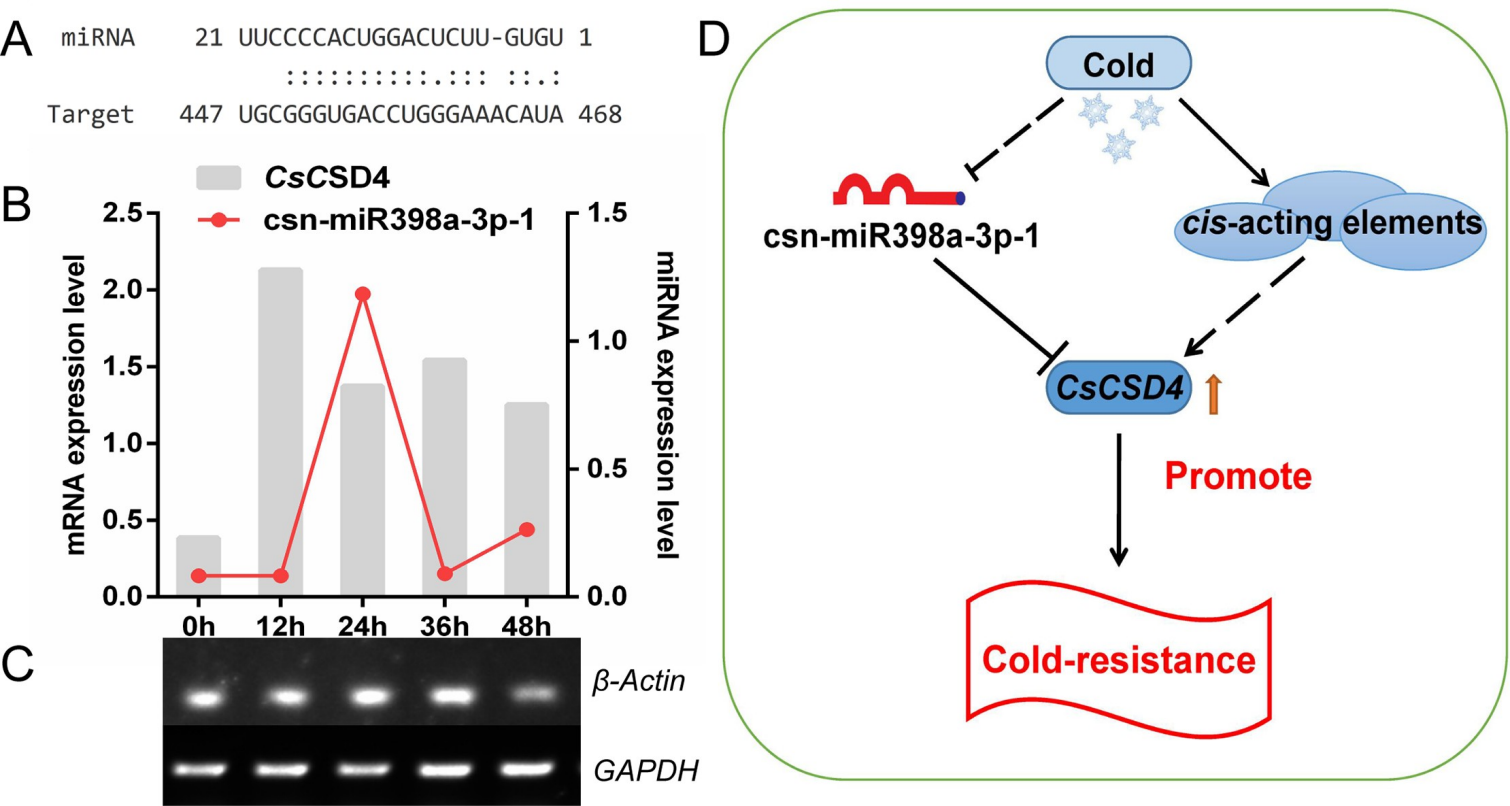
**Csn-miR398a-3p-1 and *CsCSD4* aligned fragments (A)**, **expression trends of *CsCSD4* and csn-miR398-3p-1 under cold stress (B), the electrophoretogram of *β-Actin* and *GAPDH* under cold treatment (C), a model of the expression of CsCSD4 regulated by csn-miR398a-3p-1 and *cis*-acting elements (D).** Arrows: positive regulation; blunt-ended arrows: inhibition; dotted lines: untested.

To step further understanding post-transcriptional regulation of *CsSOD* genes, we also detected the expression levels of predicted target miRNAs by triggering *CsSOD* genes cleavage under mentioned above treatments (S1 Fig). Finally, we found that the expression trend of csn-miR398-3p-1 just the opposite to *CsCSD4* under cold treatment (Fig 7B).

## Discussion

Acting as the first line of defense in antioxidant system, SODs play significant roles in catalyzing the dismutation of superoxide free radicals and protecting plant cells from oxidative damage [7]. Thus, analysis of all the members of *CsSOD* was important for provide a certain basis to understand and improve stress resistance in tea plants. The publication of tea plant genome data makes it possible to identify all *CsSOD* genes at the whole-genome level. In this research, we identified a total of 10 *CsSOD* genes based on tea plant genome, including 7 *CsCSDs*, 2 *CsFSDs*, and 1 *CsMSD*. Previous research found that there were 7 *CSDs*, 2 *FSDs* and 2 *MSDs* in *P*. *trichocarpa* [14]; 6 *CSDs*, 2 *FSDs* and 4 *MSDs* in *M*. *acuminata* [18]; 5 *CSDs*, 2 *FSDs* and 2 *MSDs* each in *G*. *raimondii* and *G*. *arboretum* [19]; 10 *CSDs*, 4 *FSDs* and 4 *MSDs* in *G*. *hirsutum* [20]; 4 *CSDs*, 1 *FSD* and 1 *MSD* in *L*. *kaempferi *[23]; 6 *CSDs*, 2 *FSDs* and 2 *MSDs* in *V*. *vinifera* [9]. The proportion of three types of *SOD* members in different plants could reflecting *CSD* genes are usually the most abundant *SOD* genes in different plants. In the analysis of potential protein interaction, we found that CsSODs with their “STRING proteins” in *A*. *thaliana* were just subdivided in same subgroups (Fig 1), indicating that different SOD members in same subgroup were highly homologous.

### Diversity of intron number in *CsSOD* gene family

In eukaryotes, gene structure generally contain exons and introns, and are divided into intron-containing genes and intronless genes according to the presence or absence of introns [61]. It is generally believed that the number of introns is closely related to the complexity of the eukaryote’s genome, and most eukaryotes have two or more introns [62]. In our study, we found that the intron numbers of 10 *CsSOD* genes were quite different, which varied between 0 (*CsCSD6*) and 10 (*CsFSD1*), notably, *CsCSD6* has no intron and its gene length was significantly shorter than that of the intron-containing *CsSOD* genes, indicating that the length of the exons was limited to a shorter range than that of the intron [63]. However, the intron numbers of 7 *AtSOD* genes varied from 5 to 8, with highest intron number in *AtMSD1* and lowest intron number in *AtFSD2*. These findings in accord with previous view that there were no similar *SOD* genes structures showed in different species [7]. Several lines of evidence suggest that structural divergences perhaps attribute to three main mechanisms: exon/intron gain/loss, exonization/pseudoexonization and insertion/deletion, which bring about orthologous genes evolving different number of intron, each of which contributed underlying structural divergences were different. And, structure divergences occurred proportionally to evolutionary time [64]. We hypothesize that various intron numbers in *CsSOD* genes may be caused by interaction of the three mechanisms in the course of long-term evolution. Intron is beneficial to species evolution, increasing the length of gene, the recombination frequency between genes, and has the function of regulation; while intronless gene has no advantage for species evolution and recombination [65]. By lateral comparison, it was showed that the expression of *CsCSD6* in tea plant presented at a relative low level among *CsCSD* genes (Fig 5). We inferred that this may be due to the simpler structure of *CsCSD6* genes and it is an older gene. This is consistent with the results of studies on intronless genes in *O*. *sativa* and *A*. *thaliana* [66].

### *CsSOD* genes play key roles in response to stress-resistance in tea plant

Excess ROS caused by abiotic stresses like cold and drought could pose threats to tea yield. SODs involved in scavenging ROS caused by several abiotic stresses in plant [10]. However, the specific responsiveness for cold, drought stress and GA_3_, MeJA stimuli in each *CsSOD* genes still unknown. Thus, the qRT-PCR detection could provide pivotal chew for understanding the potential functions of *CsSOD* genes under these stresses. In this study, the expression levels of most *CsSOD* genes revealed dynamic trends at certain timing from 0 h to 48 h under abiotic stresses (cold and drought), except for *CsFSD2* and *CsMSD1* when treated by drought. Evidence is accumulating that *SODs* hammer at overcome cold and drought stresses in *S*. *lycopersicum* [67], *G*. *hirusutm* [20], *Brassica juncea* and *B*. *rapa* [24]. It was also reported that increased expression of squash *SOD* genes along with increased activity of the SOD involved in detoxifying ROS in response to these stresses [68]. In our study, functions of *cis*-acting elements about cold- and drought-responsiveness exist in over half member promoters. However, drought-related elements have not been found in *CsFSD2* and *CsMSD1* and their expression levels were relatively stable under drought treatment. It speculated that *CsFSD2* and *CsMSD1* may not respond to drought stress. Furthermore, one study by over expressing *CSD* genes of transgenic *N*. *tabacum* showed that plants damage caused by drought stress has been mitigated to some extent [13]. In the present study, we found that *CsCSD* genes revealed remarkably expression change compared to *CsFSD1*, *CsFSD2*, and *CsMSD1* under drought stress, this observation was similar to the expression of *SOD* gens in other plants [18, 20, 69], and indicated that *CsCSD* genes may play primary roles in drought-resistance in tea plant. In addition, under cold stress, the expression trends of most Cs*SOD* genes expect for *CsFSD2* were up-regulated comparing with 0 h at different stages. These results indicated that *CsSOD* genes could respond to cold and drought signal and adopt different strategies at distinct stages.

Studies have shown that phytohormone could activate signal transduction pathway bringing about plant stress responses [70]. For instance, it has been found that the suppression of GA_3_ signalling is a general response to abiotic stress in *A*. *thaliana* [71]; in tobacco, GA_3_ signaling pathway could regulate ROS scavenge enzyme, such as SOD, peroxidase (POD) and catalase (CAT) to enhance the susceptibility of pathogen infection by overexpressing *GhMPK11* [72]. In addition, MeJA responses to biotic and abiotic stresses, which has been widely used to investigate jasmonate signaling pathways and activate defense system [73]. In our research, we have found GA-related elements exist in *CsCSD1*, *CsCSD3*, *CsCSD4*, *CsCSD5*, *CsCSD6*, *CsFSD1*, and *CsFSD2* promoters, MeJA-related elements exist in *CsFSD2*, *CsMSD1*, *CsCSD2*, *CsCSD3*, *CsCSD5*, and *CsCSD7*. These genes were significant response to exogenous GA_3_ and MeJA stimuli. We conjecture that exogenous GA_3_ and MeJA could act as signal factors in stress-resistance, and then stimulate the transcript level of *CsSOD* genes at different stages in tea plant.

### Csn-miR398a-3p-1 negatively regulated the expression of *CsCSD4* may be a crucial regulatory mechanism in tea plant under cold stress

Previous studies have explored the relationships between miRNA and plant stress-responsive, among them, miR398 was known as related to stress regulation and regulates plant responses to a series of stresses [74]. Till now, target genes of miR398, including *CSD1*, *CSD2*, *CCS1* have been found in many plants [75]. In *A*. *thaliana*, chilling resistance could occurs via negatively regulates miR398 expression and the expressions of *AtCSD1* and *AtCSD2* was induced accordingly [76]; in *Triticum aestivum*, the expression of miR398 was inhibited by cold stress and *CSD* gene was up-regulated synchronously [77]; in *O*. *sativa*, the expressions of miR398 and its targets (*OsCSD1* and *OsCSD2*) were inhibited and increased under cold stress, respectively [78]. In the present study, we perform the modification 5’ RLM-RACE experiment to verified the putative regulatory miRNAs of *CsSODs*. And we found that the cleavage site outside the complementary regions between csn-miR164-1 and *CsCSD4*, csn-miR398a-3p-1 and *CsCSD7*, and only found csn-miR398a-3p-1 directly cleaves *CsCSD4*. This result accord with previous views that many prediction tools of plant miRNA target exist a large number of false positive in non-Arabidopsis species [79]. The qRT-PCR experiment showed that the expression level of *CsCSD4* gene under cold stress was increased after 0 h as a whole. But from 12 h to 48 h, the expression revealed a “inverted W shape” trend. It speculated that the expression of *CsSOD* gene may regulated by multiple regulatory factors. To understand the regulation of *CsSOD* genes in post-transcriptional level, we detected the expression level of regulatory miRNAs of *CsSOD* genes. Finally, we only found that the expression trends of *CsCSD4* and csn-miR398a-3p-1 from 0 h to 48 h under cold stress were just reverse. It suggests that csn-miR398a-3p-1 may be a key regulatory factor of *CsCSD4* expression. Although csn-miR164-1 was also predicted regulatory miRNA of *CsCSD4*, but we not found cleavage sites in their complementary region. Similar case occurs between *CsCSD7* and csn-miR398a-3p-1. These maybe due to low psRNATarget screening criteria, which can reduce false positive by increasing screening standards. On the basis of these results, we proposed a hypothetical model to explain potential regulatory factor affecting the expression of *CsCSD4* under cold stress (Fig 7D). *Cis*-acting elements harbored cold-related function could respond to cold signal and promote *CsCSD4* expression at transcriptional level; then csn-miR398-3p-1 inhibited *CsCSD4* expression at post-transcriptional level. However, with these two regulatory factors, the expression of *CsCSD4* revealed a up-regulated trend under cold stress and devote in enhanced cold-resistance in tea plant eventually.

## Conclusions

A systematic analyses and research about *SOD* gene family in tea plant have conducted. We identified 10 *CsSOD* genes in total, including 7 *CsCSDs*, 2 *CsFSDs* and 1 *CsMSD*. Physico-chemical characteristic, phylogenetic classification, conserved motifs and potential protein interaction analyses about CsSOD proteins were carried out. Exon-intron structures and CUB pattern about *CsSOD* genes were also examined. In addition, on the basis of *cis*-acting elements, the expression profiles of *CsSOD* genes were detected by qRT-PCR under cold, drought, exogenous GA_3_ and MeJA treatments. The results showed that *CsSOD* genes play vital roles in regulating responses to these treatments. Moreover, we also detected the expression levels of predicted regulatory miRNAs of *CsSOD* genes, and we found that the expression trends of *CsCSD4* and csn-miR398a-3p-1 were just reverse under cold stress. The modification 5’ RLM-RACE experiment was performed and validated that *CsCSD4* was cleaved by csn-miR398a-3p-1. We conjecture csn-miR398a-3p-1 negatively regulated the expression of *CsCSD4* may be a crucial regulatory mechanism in tea plant under cold stress. This work provides a certain basis for the studies about stress resistance in tea plants, even provide insight into comprehending the classification, evolution, diverse functions and influencing factors of expression pattern for *CsSOD* genes.

## Supporting information

S1 Fig The expressions of regulatory miRNAs of CsSODs under four treatments. (TIF)

S2 Fig The electrophoretogram of modification 5’ RLM-RACE amplification of cleavage fragments of *CsCSD4*, *CsCSD7*, and *CsFSD2*. 1 to 5 represent fragments of *CsCSD4* cleaved by miR164-1 and csn-miR398a-3p-1, *CsCSD7* cleaved by novel-miR54 and csn-miR398a-3p-1, and *CsFSD2* cleaved by miR166d-5p-1, respectively. M represents Trans 2K DNA Marker (TransGen, Beijing, China).
(TIF)

S1 Table List of the SOD protein sequences in tea plant. (XLSX)

S2 Table The relative synonymous codon usage of *CsSOD* genes. (XLSX)

S3 Table The functions of *cis-*acting elements in *CsSOD* promoters. (XLSX)

S4 Table The predicted regulatory miRNAs of *CsSOD* genes. (XLSX)

S5 Table Specific primers of *CsSOD* genes and reference genes for qRT-PCR. (XLSX)

S6 Table The expression value of *CsSOD* genes and their predicted regulatory miRNAs under treatments by qRT-PCR. (XLSX)
