# Supplementary figures and images for: Genome-wide investigation of superoxide dismutase (SOD) gene family and their regulatory miRNAs reveal the involvement in abiotic stress and hormone response in tea plant (Camellia sinensis)
Source: PLoS One. 2019 Oct 10;14(10):e0223609. doi: 10.1371/journal.pone.0223609 (PMC6786557; doi:10.1371/journal.pone.0223609)

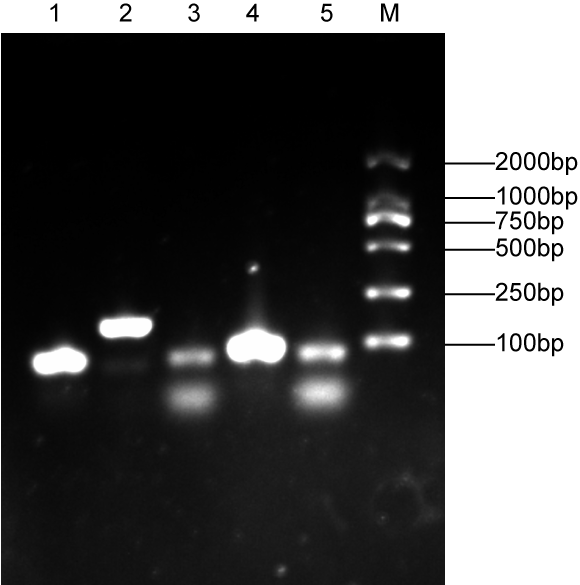

Supplement: S2 Fig — 1 to 5 represent fragments of CsCSD4 cleaved by miR164-1 and csn-miR398a-3p-1, CsCSD7 cleaved by novel-miR54 and csn-miR398a-3p-1, and CsFSD2 cleaved by miR166d-5p-1, respectively. M represents Trans 2K DNA Marker (TransGen, Beijing, China). (TIF) [file pone.0223609.s002.tif]
